# Artificial intelligence for bone metastases: a systematic review and meta-analysis of diagnostic and prognostic performance

**DOI:** 10.3389/fonc.2026.1915919

**Published:** 2026-08-24

**Authors:** Luisana Sisca, Mariam Grazia Polito, Emy Sisca, Giuseppe Francesco Papalia, Francesco Vilardi, Fabio Venuti, Alessio Cortellini, Marianna Silletta, Roberta Scafetta, Silvia Calagna, Michele Iuliani, Silvia Cavaliere, Sonia Simonetti, Bruno Vincenzi, Giuseppe Tonini, Francesco Pantano

**Affiliations:** 1 Medical Oncology, Fondazione Policlinico Universitario Campus Bio-Medico, Rome, Italy; 2 Department of Medicine and Surgery, Università Campus Bio-Medico di Roma, Rome, Italy; 3 Department of Translational Research, Institut Curie, Paris, France; 4 Community Oncology Unit, Azienda Sanitaria Locale (ASL) Latina, Casa della Salute (CDS) Aprilia, Sapienza University of Rome, Rome, Italy; 5 Department of Mechanical, Energy and Management Engineering (DIMEG), University of Calabria, Rende, Italy; 6 Oncological Orthopedics Department, Istituti Fisioterapici Ospitalieri (IFO), Istituto di Ricovero e Cura a Carattere Scientifico (IRCCS) Regina Elena National Cancer Institute, Rome, Italy; 7 Department of Surgery and Cancer, Imperial College London, London, United Kingdom

**Keywords:** artificial intelligence, bone metastases, deep learning, machine learning, meta-analysis, radiomics, systematic review

## Abstract

Bone metastases are a major complication of advanced solid tumors and are associated with substantial morbidity and reduced survival. Imaging plays a central role in detection and monitoring, yet conventional modalities and qualitative risk scores remain limited by suboptimal specificity and inter-reader variability. Artificial intelligence (AI) has emerged as a potential tool to improve diagnostic accuracy and prognostic assessment. We performed a systematic review and meta-analysis of studies published between January 2008 and January 2026 evaluating AI-based models for the diagnosis and/or prognosis of bone metastases. PubMed/MEDLINE, Scopus, and Web of Science were searched. Studies reporting quantitative performance metrics were included. Logit-transformed area under the curve (AUC) values were pooled using a random-effects model with restricted maximum likelihood estimation. Twenty-two studies met the eligibility criteria, encompassing highly heterogeneous datasets ranging from small single-center cohorts to large population-based registries and multicenter imaging databases. Most were retrospective and single-center. The overall AUC was 0.911 (95% CI 0.868–0.940), with substantial heterogeneity (I² = 98.8%) Radiomics-based models showed consistently high performance with minimal heterogeneity, after group stratification, whereas clinical-only models demonstrated lower discriminative ability. Prognostic studies were heterogeneous and were synthesized narratively. AI-based models demonstrate high diagnostic performance for bone metastases across imaging modalities. However, methodological variability and limited external validation currently restrict clinical translation, underscoring the need for prospective multicenter studies. Most included studies were retrospective, single-center investigations with limited external validation. Therefore, despite the encouraging diagnostic performance, prospective multicenter studies and standardized reporting remain necessary before routine clinical implementation. Unlike previous systematic reviews, this study provides a quantitative synthesis of diagnostic performance together with a structured appraisal of methodological quality and clinical translational readiness. This systematic review and meta-analysis was conducted in accordance with the PRISMA 2020 statement and prospectively registered (CRD420261350260).**Systematic review registration:** https://www.crd.york.ac.uk/PROSPERO/view/CRD420261350260, identifier CRD420261350260.

## Introduction

Bone metastases are among the most frequent and debilitating manifestations of advanced malignancy, particularly in breast, prostate, and lung cancers (1). Their occurrence is closely associated with skeletal-related events such as pathological fractures, spinal cord compression, and intractable pain, all of which substantially impair quality of life and shorten survival. Although systemic oncologic therapies have advanced considerably, the timely identification of skeletal involvement and accurate estimation of mechanical instability remain persistent clinical challenges (2).

Imaging constitutes the cornerstone of diagnosis and surveillance. Bone scintigraphy continues to be widely used for its sensitivity, yet its limited specificity frequently results in false-positive findings. Computed tomography offers excellent anatomical detail but may fail to detect early marrow infiltration (3). Magnetic resonance imaging provides superior sensitivity for marrow disease, although its availability and cost can restrict routine use. Positron emission tomography achieves high diagnostic accuracy but remains resource-intensive and not universally accessible. Across modalities, image interpretation is inherently dependent on reader expertise and is subject to variability (4).

Risk assessment tools such as Mirels’ score and the Spinal Instability Neoplastic Score were developed to assist clinical decision-making, particularly regarding fracture risk. While valuable, these systems are largely qualitative and demonstrate only moderate discriminative performance, occasionally leading to both overtreatment and undertreatment (5).

Against this background, artificial intelligence has emerged as a promising strategy to address some of these limitations. Radiomics enables extraction of high-dimensional quantitative features from standard imaging studies, capturing subtle patterns that may escape visual assessment. Deep learning architectures, including convolutional neural networks and transformer-based models, can learn complex image representations directly from data without predefined feature engineering (6). Furthermore, AI frameworks offer the possibility of integrating imaging with clinical, pathological, and even genomic information, thereby reflecting the biological heterogeneity of metastatic disease. Several systematic reviews published in 2025–2026 have summarized AI applications for bone metastases, in some cases including nearly 100 primary studies across heterogeneous imaging modalities and clinical applications, such as the review by van der Linden et al. (7) and the diagnostic meta-analysis by Tao et al. (8) Unlike previous broad reviews, our objective was not to maximize the number of included studies but to maximize methodological comparability for quantitative synthesis. By restricting inclusion to studies reporting standardized performance metrics suitable for meta-analysis and structured quality assessment, we aimed to provide a more reliable estimate of AI diagnostic performance and a critical evaluation of its current clinical translational readiness. However, these reviews generally included highly heterogeneous AI applications across multiple imaging modalities and clinical settings, often without restricting quantitative synthesis to methodologically comparable studies. Consequently, although they provided comprehensive overviews of the field, the methodological heterogeneity limited detailed quantitative comparisons of diagnostic performance and clinical readiness. In contrast, the present review was specifically designed to evaluate studies reporting quantitative diagnostic or prognostic performance metrics suitable for structured methodological quality assessment and, where appropriate, quantitative synthesis. Consequently, more restrictive eligibility criteria were applied to ensure methodological comparability across studies included in the meta-analysis.

Accordingly, the present systematic review and meta-analysis was designed to extend the existing literature by providing a quantitative synthesis of diagnostic performance, a structured methodological quality assessment using validated tools, subgroup analyses according to AI methodological domains, and a critical appraisal of the current translational readiness of AI applications for bone metastases (9).

## Methods

This systematic review and meta-analysis was conducted in accordance with the PRISMA 2020 statement and prospectively registered (CRD420261350260).

### Search strategy

**Figure 1:**
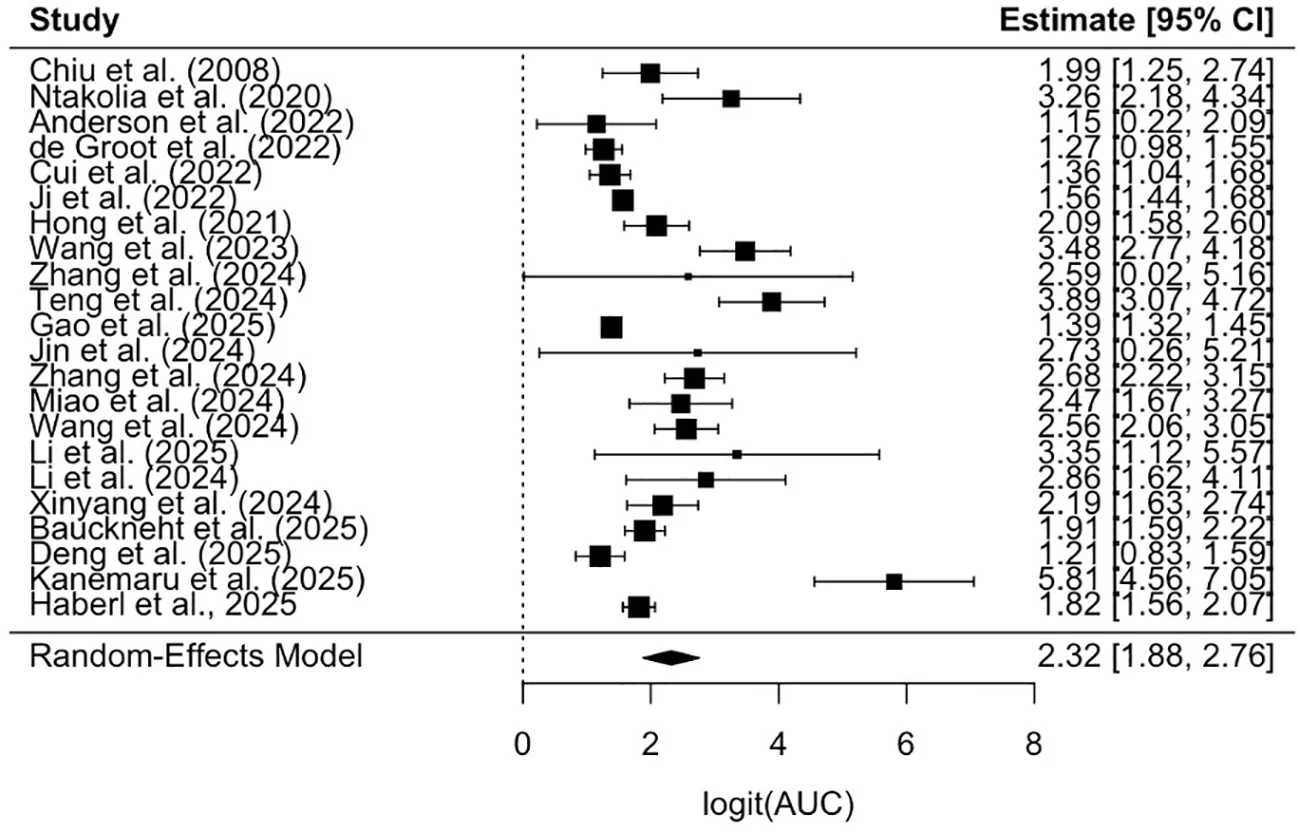
PRISMA 2020 flow diagram summarizing the study identification and selection process for the systematic review and meta-analysis on AI in bone metastases.

A comprehensive literature search was conducted in PubMed/MEDLINE, Scopus, and Web of Science to identify studies evaluating artificial intelligence (AI)-based models for the diagnosis and prognosis of bone metastases. The search covered publications from January 2008 to January 2026. Two complementary search strategies were developed by combining controlled vocabulary (Medical Subject Headings [MeSH], where applicable) and free-text terms related to artificial intelligence, machine learning, deep learning, radiomics, bone metastases, diagnosis, and prognosis, using the Boolean operators “AND” and “OR”. The complete search strategies are reported in **Supplementary Table 1**. The search strategy was developed *a priori* and adapted to the syntax requirements of each database while maintaining equivalent search concepts across all databases. In addition, the reference lists of all included articles and relevant review papers were manually screened to identify any additional eligible studies. The study selection process is summarized in **Figure 1**.

### Eligibility criteria

Eligibility criteria were defined according to the Population, Intervention, Comparator, Outcomes, and Study design (PICOS) framework. Studies were considered eligible if they met the following criteria: (P) adult patients with suspected or confirmed bone metastases from solid tumors; (I) evaluation of artificial intelligence-based models, including machine learning, deep learning, or radiomics approaches, for diagnostic or prognostic purposes; (C) conventional diagnostic approaches, clinical prediction models, expert assessment, or no comparator when model performance was evaluated independently; (O) quantitative performance metrics, including area under the receiver operating characteristic curve (AUC), sensitivity, specificity, accuracy, concordance index (C-index), or equivalent validated measures; and (S) original peer-reviewed observational studies. Reviews, editorials, letters, conference abstracts without full-text articles, case reports, animal or preclinical studies, and studies that did not report quantitative performance metrics were excluded.

### Study selection and data extraction

Two reviewers (L.S. and M.G.P.), both experienced in systematic reviews and oncological research, independently screened all retrieved records by title and abstract. Full texts of potentially eligible articles were subsequently assessed according to the predefined eligibility criteria. Any disagreements were resolved through discussion and consensus. Screening and eligibility assessment were performed independently and in duplicate to minimize selection bias.

Extracted variables included first author, publication year, country, study design, cancer type, number of patients, imaging modality, anatomical site of bone metastases (when reported), lesion characteristics (osteolytic, osteoblastic, mixed, when available), number of metastatic lesions (when reported), AI methodology, validation strategy (internal, external, or cross-validation), study objective, reference standard, outcome measures, and reported performance metrics. Particular attention was paid to variables potentially affecting model generalizability, including external validation, imaging modality, study design, and AI methodological domain.

### Quality assessment

Methodological quality was assessed for all included studies according to their study design. Diagnostic studies were evaluated using the Quality Assessment of Diagnostic Accuracy Studies-2 (QUADAS-2) tool, whereas prognostic studies were assessed using the Prediction model Risk Of Bias ASsessment Tool for Artificial Intelligence (PROBAST+AI), which is specifically designed to evaluate the risk of bias and applicability of AI-based prediction models. Two reviewers independently performed the quality assessment according to the published guidance for each tool. Any disagreements were resolved through discussion until consensus was reached. Risk-of-bias judgments (low, high, or unclear risk) were assigned for each domain of the respective assessment tool, and the results are summarized in **Tables 1**, **2**. Rather than generating an overall quality score, domain-specific judgments were used to identify recurrent methodological weaknesses across studies, thereby informing the interpretation of pooled results. Studies were judged at higher risk of bias mainly because of retrospective study design, limited external validation, small sample size, incomplete reporting of model development or calibration procedures, and potential overfitting. Studies judged as presenting some concerns generally lacked sufficient methodological information regarding patient selection procedures or reference standard implementation to support a low-risk judgment, although no major methodological flaws were identified. Conversely, studies classified as high risk typically combined retrospective single-center designs with limited external validation, incomplete reporting of model development or calibration, and relatively small datasets, thereby increasing the potential for overfitting and limiting model generalizability. Consistent with the recommendations accompanying QUADAS-2 and PROBAST+AI, no overall numerical quality score was assigned. Instead, individual domain-level judgments were used to identify the principal methodological limitations of each study.

**Table 1:** Methodological characteristics, AI approaches, input data, validation strategies, and cancer types of the included studies (n = 22).

Variable	n (%)
Study objective	
Diagnostic/detection of bone metastasis	15 (68.2)
Prognostic/survival or progression prediction	6 (27.3)
Combined diagnostic and prognostic	1 (4.5)
Study design	
Retrospective development cohort	22 (100)
Multicenter cohort	8 (36.4)
External validation performed	10 (45.5)
Prospective validation cohort	1 (4.5)
AI methodology*	
Machine learning	10 (45.5)
Deep learning	5 (22.7)
Radiomics	4 (18.2)
Transformer-based	1 (4.5)
Large language model	1 (4.5)
Generative AI	1 (4.5)
Primary input data*	
Clinical variables	11 (50.0)
Laboratory/serum biomarkers	5 (22.7)
CT	4 (18.2)
Bone scintigraphy	3 (13.6)
MRI	2 (9.1)
PET/CT	2 (9.1)
Pathology/whole-slide images	2 (9.1)
Radiology reports	1 (4.5)
Validation strategy	
Internal validation only	16 (72.7)
External validation	6 (27.3)
Cancer type	
Prostate cancer	11 (50.0)
Breast cancer	4 (18.2)
Lung cancer	3 (13.6)
Mixed solid tumors/mixed bone metastases	2 (9.1)
Kidney cancer	1 (4.5)
Cancer of unknown primary	1 (4.5)

* Categories are not mutually exclusive; therefore, percentages may exceed 100%.

**Table 2:** Risk of bias assessment of included diagnostic studies using the QUADAS-2 tool across four domains: patient selection, index test, reference standard, and flow and timing.

Study	Patient selection	Index test	Reference standard	Flow and timing	Overall
Chiu et al. (2008) (10)	Some concerns	Low	Low	Low	Some concerns
Ntakolia et al. (2020) (11)	Some concerns	Low	Low	Low	Some concerns
Ji et al. (2022) (12)	High	High	Unclear	Low	High
Hong et al. (2021) (13)	Some concerns	Low	Low	Low	Some concerns
Wang et al. (14) (14)	High	High	Low	Low	High
Zhang et al. (2024) (15)	Low	Low	Low	Some concerns	Some concerns
Teng et al. (2024) (16)	High	High	Unclear	Low	High
Gao et al. (17) (17)	Low	Low	Low	Some concerns	Some concerns
Li et al. (18) (18)	High	High	Unclear	Low	High
Li et al. (2024) (19)	Low	Low	Low	Some concerns	Some concerns
Xinyang et al. (2024) (20)	High	High	Unclear	Low	High
Bauckneht et al. (2025) (21)	Some concerns	Low	Some concerns	Low	Some concerns
Haberl et al. (2025) (22)	High	High	Unclear	Low	High
Wang et al. (2024) (14)	High	High	Unclear	Low	High
Kanemaru et al. (2025) (23)	Some concerns	Low	Low	Low	Some concerns

High-risk judgments were primarily assigned because of retrospective single-center patient selection, absence of external validation, incomplete or unclear reference standards, and insufficient reporting of model development or validation procedures, all of which may increase the risk of overfitting and limit generalizability. Studies classified as "Some concerns" generally showed adequate methodology but lacked sufficient information in one or more QUADAS-2 domains to support a low-risk judgment.

### Diagnostic studies were evaluated using QUADAS-2, while prognostic models were assessed with PROBAST.

Logit transformation was applied because AUC values are bounded between 0 and 1, and transformation improves the approximation to normality required for random-effects meta-analysis. For quantitative synthesis, logit-transformed AUC values were pooled using a random-effects model with restricted maximum likelihood (REML) estimation. All statistical analyses were performed using R (R Foundation for Statistical Computing, Vienna, Austria), and random-effects meta-analysis was conducted using the *metafor* package. When 95% confidence intervals were available, standard errors were derived accordingly; otherwise, variance estimates were approximated using established methods for AUC meta-analysis. Statistical heterogeneity was assessed using Cochran’s Q test and quantified with the I² statistic. Subgroup analyses were prespecified to explore potential sources of heterogeneity and were conducted according to primary methodological domain (clinical-only models, nuclear imaging–based models, and radiomics-based models). Deep learning models applied to conventional cross-sectional imaging (CT or MRI) without structured radiomics extraction were not pooled separately due to limited methodological homogeneity and were therefore included in the overall analysis. Although radiomics-based approaches and end-to-end deep learning models rely on different feature extraction strategies, both ultimately address the same clinical question, namely the discrimination of patients or lesions with versus without bone metastases. Therefore, despite methodological differences, they share a common diagnostic endpoint and a directly comparable summary performance measure (AUC), making an overall synthesis appropriate for estimating global AI diagnostic performance. Accordingly, the primary unit of synthesis in the present meta-analysis was the reported diagnostic discrimination rather than the underlying computational architecture. To account for methodological diversity, prespecified subgroup analyses were subsequently performed whenever sufficient homogeneity was present. This approach is consistent with previous methodological recommendations indicating that studies addressing the same clinical question may be quantitatively synthesized despite differences in AI architecture, provided that comparable outcome measures are analyzed and clinical heterogeneity is explicitly acknowledged.

Given differences in clinical tasks, imaging modalities, and outcome definitions, pooled AUC estimates were interpreted as global indicators of discriminative performance rather than direct clinical benchmarks. Prognostic studies were summarized narratively due to heterogeneity in endpoints and reporting. Sensitivity and specificity were extracted whenever reported and considered during the qualitative synthesis. However, quantitative pooling of these metrics, including hierarchical summary receiver operating characteristic (HSROC) and bivariate random-effects analyses, was not feasible because fewer than half of the included studies reported sufficient two-by-two contingency data (true positives, false positives, true negatives, and false negatives). Formal assessment of publication bias was not performed for subgroup analyses due to the limited number of studies per category, which reduces the reliability of funnel plot–based methods.

## Results

Twenty-two studies published between 2008 and 2026 (10–32). The main characteristics of the included studies, including AI methodologies and reported performance metrics, are summarized in **Table 3**. Of the 22 included studies, 15 focused primarily on diagnostic applications, 6 evaluated prognostic models, and 1 addressed both diagnostic and prognostic objectives. Regarding AI methodology, image-based approaches (radiomics and deep learning) accounted for the majority of studies, while a smaller proportion investigated machine-learning models based exclusively on clinical or laboratory variables. CT and MRI were the most frequently evaluated imaging modalities, followed by PET/CT, bone scintigraphy/SPECT, and multimodal imaging. Lung, prostate, and breast cancer represented the most commonly investigated tumor types. The majority were retrospective and conducted in single institutions. Computed tomography was the most commonly employed imaging modality, although MRI, PET/CT, SPECT, and multimodal approaches were also represented.

**Table 3:** Characteristics of the included studies, including cancer type, study design, sample size, target lesion or anatomical site, AI methodology, validation strategy, primary objective, and reported diagnostic or prognostic performance.

Study	Cancer type		Study design	Sample size	Target lesion / anatomical site	Input data / Imaging	AI approach	Validation	Primary objective	Main performance
Chiu et al. (2008) (10)	Prostate cancer		Retrospective, single-center	111 patients	Skeletal metastases	Clinical variables (age, PSA) + bone scintigraphy reference	Artificial Neural Network (ANN)	Internal	Predict skeletal metastases	AUC 0.88; Sens 87.5%; Spec 83.3%
Hong et al. (2021) (13)	Mixed (bone islands vs osteoblastic metastases)		Retrospective, bicentric	241 patients (177 training, 64 external test)	Sclerotic bone lesions	Contrast-enhanced CT	CT radiomics + Random Forest	10-fold CV + external validation	Differentiate bone islands from osteoblastic metastases	External AUC 0.96; Accuracy 86%
Wang et al. (2023) (14)	Mixed cancers with spinal metastases		Retrospective	941 patients	Spinal metastases	MRI	CNN + Softmax classifier	Internal	Diagnose spinal metastases	Accuracy 96.5%; AUC 0.97
de Groot et al. (2023) (29)	Extremity bone metastases		Retrospective temporal validation	406 patients	Long-bone metastases	Clinical/laboratory variables	SORG machine learning algorithm	Temporal external validation	Predict 90-day and 1-year survival	AUC 0.78 (90-day), 0.75 (1-year)
Teng et al. (2024) (16)	NSCLC		Retrospective + external + prospective validation	664 training; 113 external; 316 prospective	Bone metastases	Serum biomarkers	Random Forest (best model)	Internal + external + prospective	Early diagnosis of bone metastases	External AUC 0.97
Bauckneht et al. (2024/2025) (21)	Hormone-sensitive prostate cancer		Retrospective multicenter	267 patients (448 lesions)	PSMA-positive bone lesions	[18F]PSMA-1007 PET/CT + clinical variables	Radiomics/ensemble ML (BUMP score)	10-fold internal + external validation	Distinguish benign uptake from bone metastases	AUC 0.87; External AUC 0.92
Gao et al. (2025) (17)	Breast cancer		Retrospective (SEER)	24,584 patients	Bone metastases	Clinical variables	ML (MLPNN, RF, GBM, LR, AdaBoost)	Internal validation	Predict bone metastases	Best AUC 0.808 (MLPNN)
Deng et al. (2025) (28)	Primary and metastatic bone tumors		Secondary analysis of RCT (PARITY)	604 patients	New distant metastases	Clinical variables	LightGBM + SHAP	Internal hold-out validation	Predict 1-year metastasis and mortality	AUC 0.77 (metastasis); 0.78 (mortality)
Wang et al. (2025) (31)	Breast cancer (murine model)		Preclinical longitudinal study	260 serial μCT scans	Osteolytic bone lesions	Sequential μCT	Spatial-temporal neural network + FEA	Internal	Predict lesion progression and fracture risk	Dice 0.814; R² 0.955
Anderson et al. (2022) (30)		Prostate cancer	Retrospective multicenter	438 patients	Clinical variables	Multiple ML survival models (PATHFx)	Internal validation	Predict 1-, 2-, 3-, 4-, 5-, 10-year survival	AUC >0.73 across models	
Cui et al. (2022) (27)		Cancer of unknown primary	Retrospective (SEER) + external validation	1,010 + 101 external	Clinical variables	RF, GBM, DT, XGBoost	Internal + external	Predict 3-month mortality	Best RF AUC 0.796	
Ji et al. (2022) (12)		Kidney cancer	Retrospective (SEER) + external validation	71,414 + 963 external	Clinical variables	LR, NBC, DT, XGB, MLP, RF, SVM, KNN	Internal + external	Predict bone metastasis risk and prognosis	AUC 0.827 (internal); 0.912 (external prognosis)	
Jin et al. (2024) (24)		Prostate cancer	Retrospective	404 patients	Clinical + inflammatory/nutritional markers	LR, RF, NB, KNN, XGB, DT	5-fold CV + test set	Predict bone metastases	Best KNN AUC 0.939	
Li et al. (2024) (19)		Prostate cancer	Retrospective	286 patients	Clinical variables	Random Forest classifier	Internal validation	Predict progression to CRPC	AUC 0.946 (training); 0.927 (validation)	
Xinyang et al. (2024) (20)		Prostate cancer	Retrospective multicenter	348 patients	bpMRI radiomics	LR, RF, XGBoost, SVM, GBDT, AdaBoost, KNN	Internal + external	Predict bone metastases	Best XGBoost AUC 0.928 (internal); 0.921 (external)	
Zhang et al. (2024) (15)		Breast cancer	Dual-center retrospective	239 patients	Clinical blood biomarkers	LightGBM	Internal + external	Predict de novo bone metastasis	External AUC 0.908	
Kanemaru et al. (2025) (23)		Mixed cancers	Retrospective	9,559 training; 1,498 validation; 7,399 test	Unstructured radiology reports	Fine-tuned LLM	Validation + independent test	Detect progressive bone metastasis	Accuracy 97.9%	
Haberl et al. (2025) (22)		Mixed cancers	Multicenter retrospective	9,170 training; 7,472 external testing scans	Bone scintigraphy	StyleGAN2 + CNN	External multicenter	Generate synthetic scintigraphy and improve detection	Mean AUC improvement +33%	
Ntakolia et al. (2020) (11)		Prostate cancer	Retrospective	817 patients (908 bone scintigraphy images; 778 included after preprocessing)	Whole-body bone scintigraphy	Lightweight CNN (LB-FCN)	Internal	Classify bone metastases on bone scintigraphy	AUC 0.85	
Miao et al. (2024) (32)		Breast cancer	Retrospective	431 patients	CT images + clinical/body composition (subcutaneous fat index, HOG/LBP features)	Multimodal CNN + GBDT	Three-fold cross-validation	Predict bone metastasis using multimodal deep learning	AUC 0.922 (95% CI 0.843–0.964)	
Zhang et al. (2024) (15)		Prostate cancer	Retrospective	211 patients	Multiparametric MRI (T2WI, DWI, ADC) + pathology (H&E)	ResNet50 deep transfer learning + radiomics/pathomics + ML	Training/validation cohort (169/42)	Predict bone metastasis using multimodal MRI radiomics and pathomics	AUC 0.93 (95% CI 0.854–1.000)	
Li et al. (2025) (18)		Lung cancer	Retrospective	215 patients	Chest CT + digitized pathology images	Multimodal Swin Transformer (Swin-Dual Fusion)	Internal	Predict risk of bone metastasis in lung cancer	AUC 0.966; Sensitivity 88.5%; Specificity 94.4%	

**Figure 2:**
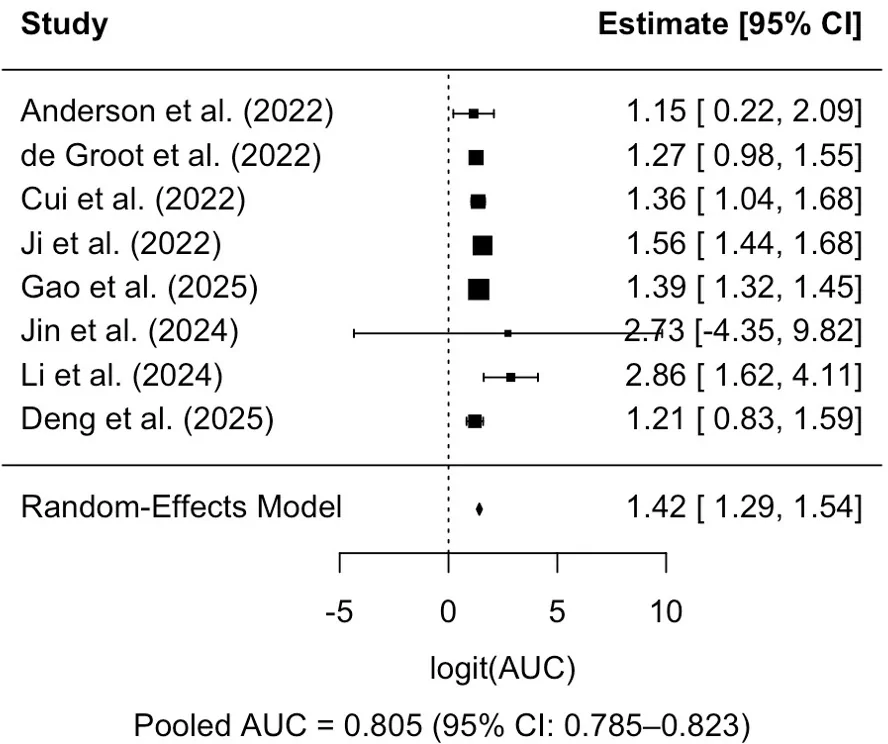
Forest plot of the overall discriminative performance of AI-based models for bone metastases based on logit-transformed AUC values The pooled estimate corresponds to an AUC of 0.911 (95% CI: 0.868–0.940). Substantial heterogeneity was observed (I² = 98.8%, τ² = 1.20, Q = 6569.7; p < 0.001).

AI methodologies were heterogeneous. Several studies employed convolutional neural networks or other deep learning architectures, including transformer-based models. Others relied on radiomics feature extraction combined with classical machine learning algorithms, while a smaller subset evaluated models based exclusively on clinical variables. External validation was reported in a minority of studies, with most relying on internal validation strategies. A quantitative summary of the methodological and clinical characteristics of the included studies, including study objectives, study design, AI methodological domains, validation strategy, input data, and cancer types, is presented in **Table 1**. The methodological quality of diagnostic studies assessed using QUADAS-2 is presented in **Table 2**. The methodological quality assessment of diagnostic and prognostic studies is summarized in **Tables 2**, **4**. Across diagnostic studies, the most frequent sources of risk of bias identified by QUADAS-2 were retrospective patient selection, limited external validation, and insufficient reporting of reference standards. In prognostic studies assessed with PROBAST+AI, the analysis domain was most commonly judged at high risk because of small sample sizes, inadequate handling of missing data, absence of external validation, and potential model overfitting. Studies classified as presenting some concerns generally lacked sufficient methodological information regarding patient selection procedures or reference standard implementation to support a low-risk judgment, although no major methodological flaws were identified. Conversely, studies judged at high risk of bias typically combined retrospective single-center designs with limited external validation, incomplete reporting of model development or calibration, and relatively small datasets, thereby increasing the potential for overfitting and limiting model generalizability. These limitations should be considered when interpreting the reported diagnostic and prognostic performance. Across diagnostic applications, the pooled AUC was 0.911 (95% CI: 0.868–0.940), with substantial heterogeneity (I² = 98.8%) (**Figure 2**). Sensitivity and specificity were extracted whenever reported in the original studies and considered during the qualitative synthesis. However, quantitative pooling of these metrics was not feasible because most studies did not provide sufficient two-by-two contingency data (true positives, false positives, true negatives, and false negatives).

**Table 4:** Risk of bias assessment of included prognostic studies using the PROBAST+AI tool across four domains: participants, predictors, outcome, and analysis. High overall risk mainly reflected retrospective study design, small sample size, lack of external validation, and potential overfitting.

Study	Participants	Predictors	Outcome	Analysis	Overall
Jin et al. (2024) (24)	High	Low	Low	High	High
Zhang et al. (2024) (15)	High	Low	Low	High	High
Miao et al. (2024) (32)	High	Low	Low	High	High
Deng et al. (2025) (28)	High	Low	Low	High	High
Anderson et al. (30) (30)	Low	Low	Low	High	High
Cui et al. (27) (27)	Low	Low	Low	High	High
de Groot et al. (2023) (29)	Low	Low	Low	Low	Low

**Figure 3:**
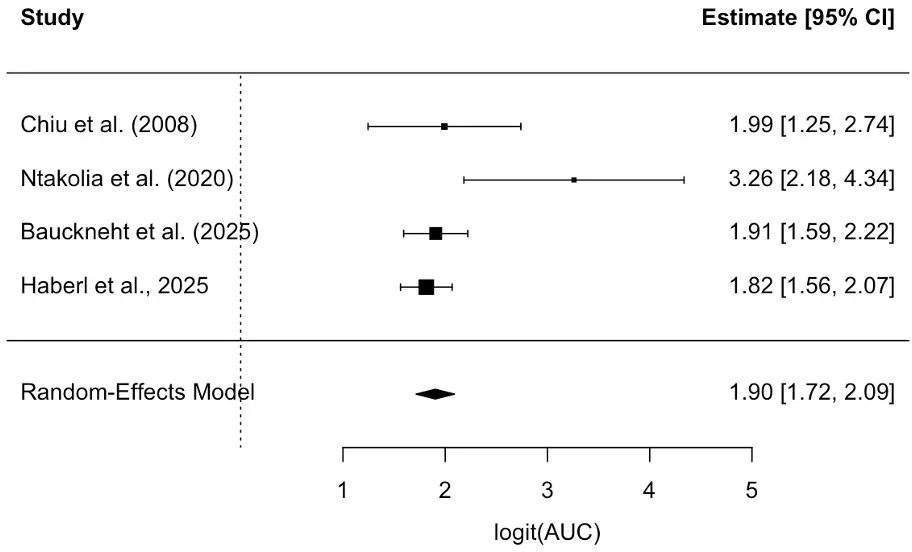
Forest plot of AI models based on clinical features. Subgroup meta-analysis of clinical-only machine-learning models (k = 8), reporting pooled logit-AUC and study-level estimates. Pooled AUC (random effects): 0.805 (95% CI: 0.785–0.823). Moderate heterogeneity was observed (I² = 44.9%, τ² = 0.009, Q = 14.7; p = 0.040).

In contrast, clinical-only models exhibited lower discriminative ability and moderate variability. Subgroup analysis of clinical-only models is presented in **Figure 3**.

**Figure 4:**
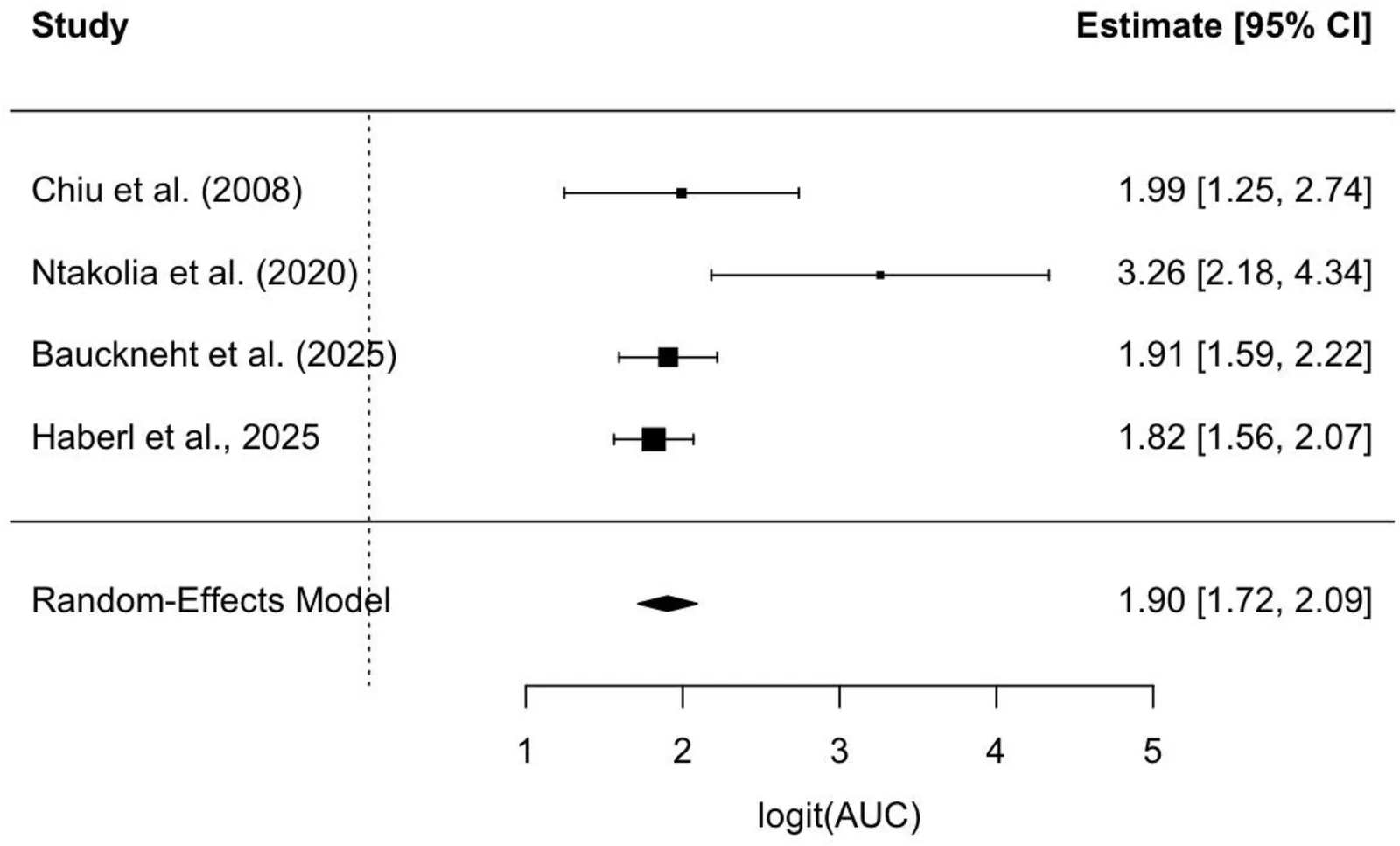
Forest plot of AI applied to nuclear imaging modalities. Diagnostic performance of AI models using PET, SPECT, or bone scintigraphy. Random-effects meta-analysis of logit-transformed AUCs (k = 4), corresponding to a pooled AUC of 0.87 (95% CI: 0.85–0.89). No significant heterogeneity was detected (I² = 0%, τ² ≈ 0, Q = 6.62; p = 0.085).

**Figure 5:**
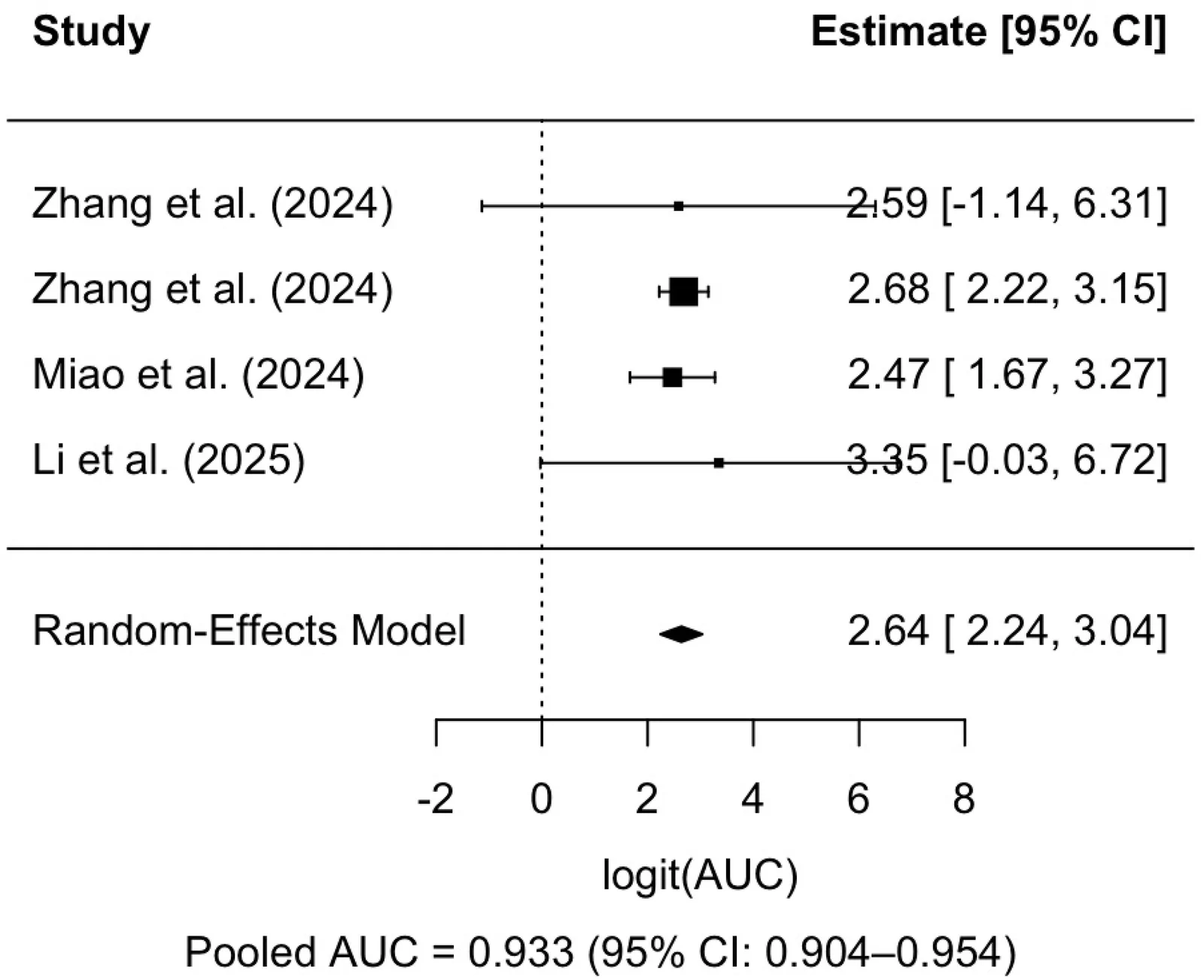
Forest plot of radiomics-based AI models derived from CT, MRI, and PET/CT. Subgroup analysis showing pooled logit-AUC and individual study estimates (k = 4). Consistently high diagnostic performance with no detectable heterogeneity (I² = 0%, τ² = 0, Q = 0.37; p = 0.95).

Importantly, subgroup analyses stratified by methodological category demonstrated markedly reduced heterogeneity, with near-zero I² in radiomics-based and nuclear imaging–based models. The pooled performance of nuclear imaging–based models is illustrated in **Figure 4**. The corresponding subgroup forest plot for radiomics-based models is shown in **Figure 5**. This pattern suggests that variability was primarily driven by between-domain differences rather than statistical instability within homogeneous methodological subgroups.

Prognostic studies addressed diverse endpoints, including early marrow alterations, lesion progression, mechanical instability, and fracture risk prediction. Risk of bias assessment of prognostic models using PROBAST+AI is summarized in **Table 4**. Differences in outcome definitions and reporting metrics precluded quantitative pooling, but available evidence suggests promising, though preliminary, performance.

## Discussion

The present synthesis indicates that AI-based models achieve consistently high discriminative performance in the detection of bone metastases across multiple imaging modalities. The pooled AUC of 0.911 (95% CI 0.868–0.940) reflects the overall discriminative performance in diagnostic modeling within this field. However, this estimate should not be interpreted as a universal clinical benchmark, given the extreme between-study heterogeneity observed in the overall analysis (I² = 98.8%). Given this substantial heterogeneity, the pooled AUC should be interpreted as a global summary indicator of performance across diverse methodological contexts rather than as a precise predictive estimate applicable to individual clinical settings (33). Although the pooled AUC indicates encouraging overall diagnostic performance, the available evidence remains dominated by retrospective single-center studies with limited external validation. Therefore, AI applications for bone metastases should currently be considered in a transitional phase between proof-of-concept development and routine clinical implementation. Although the included studies comprised both diagnostic and prognostic applications, only diagnostic studies contributed to the quantitative synthesis, whereas prognostic studies were summarized narratively because of substantial heterogeneity in endpoints and reporting. Such heterogeneity likely reflects substantial cross-domain variability, including differences in imaging modalities, AI architectures, clinical tasks (lesion-level versus patient-level analyses), and reference standards, rather than statistical instability. Although the pooled AUC provides a robust overall estimate of discriminative performance, sensitivity and specificity remain essential indicators of clinical diagnostic utility. However, these measures were inconsistently reported across the included studies, and several investigations addressed prognostic rather than diagnostic outcomes. Consequently, only a descriptive synthesis of these metrics was feasible. Future primary studies should adopt standardized reporting of diagnostic accuracy metrics, including sensitivity, specificity, and confusion matrix data, to facilitate more comprehensive evidence synthesis. Our findings should also be interpreted in the context of recently published systematic reviews. The studies included in this review also illustrate the rapid evolution of AI methodologies, ranging from conventional machine-learning algorithms and radiomics-based models to deep learning, transformer architectures, large language models, and generative AI. While imaging-based diagnostic applications appear closest to clinical translation, emerging applications such as automated report interpretation and synthetic image generation remain investigational. Recent systematic reviews have provided complementary perspectives on AI applications for bone metastases. Tao et al. performed a diagnostic meta-analysis focusing on the accuracy of AI for bone metastasis detection, whereas van der Linden et al. systematically reviewed 92 studies with particular emphasis on methodological quality, reporting guideline adherence (TRIPOD, CLAIM, and UPM), and clinical implementation. In contrast, the present review deliberately applied more restrictive eligibility criteria to include only studies reporting standardized quantitative diagnostic or prognostic performance metrics suitable for structured methodological quality assessment and quantitative synthesis. Although this approach resulted in a smaller number of eligible studies, it provided greater methodological comparability and allowed a more focused appraisal of the current translational readiness of AI models. Therefore, the present review should be considered complementary rather than an alternative to previous systematic reviews, providing a focused quantitative synthesis of methodologically comparable studies while contextualizing the maturity of current AI applications.

Notably, when analyses were stratified by methodological category, heterogeneity decreased markedly, with near-zero I² in radiomics-based and nuclear imaging–based subgroups, supporting the interpretation that variability was primarily driven by between-domain differences rather than inconsistency within homogeneous methodological strata. Compared with conventional imaging interpretation and qualitative risk scores, AI models offer several conceptual advantages. However, the absence of detectable heterogeneity in small subgroups (k = 4) should be interpreted cautiously, as statistical power to detect between-study variability is inherently limited when the number of included studies is small. Importantly, high discriminative performance does not necessarily translate into improved clinical outcomes unless AI systems demonstrate reproducibility, robustness, and meaningful impact on clinical decision-making in prospective real-world settings. They provide quantitative, reproducible outputs and can incorporate multimodal information, potentially enhancing sensitivity for subtle disease and improving risk stratification. However, discrimination metrics alone do not equate to clinical utility. Few studies reported calibration measures, decision-curve analysis, or patient-centered outcomes, limiting conclusions regarding real-world impact. Our findings are broadly consistent with recently published systematic reviews, which also reported encouraging diagnostic performance of AI for bone metastases. However, the present review extends previous evidence by providing subgroup meta-analyses according to methodological domains, structured quality assessment using validated tools, and a stronger focus on the translational readiness of current AI models.

Several limitations of both the available evidence and the present review should be acknowledged. Although the number of studies included in the quantitative synthesis was relatively small, this primarily reflects the application of predefined eligibility criteria requiring sufficiently homogeneous study designs and quantitative performance metrics suitable for meta-analysis, rather than a lack of published evidence.

The evidence base is further constrained by the predominance of retrospective, single-center studies and the limited use of external validation. Consequently, most currently available AI models should still be regarded as proof-of-concept systems rather than tools ready for widespread clinical implementation. Furthermore, although the present review primarily focused on clinical applications of artificial intelligence, one included study evaluated a preclinical murine model. This study was retained because it investigated an AI-driven prognostic approach directly related to bone metastasis biology; however, its findings should be interpreted with caution, as experimental animal models cannot fully capture the biological complexity and clinical heterogeneity of human metastatic disease. Moreover, despite subgroup analyses reducing statistical heterogeneity, clinically relevant heterogeneity persisted across cancer types, imaging modalities, AI methodologies, and intended clinical applications. Heterogeneous reference standards, variability in imaging acquisition and preprocessing, and inconsistent reporting of model development further contribute to methodological heterogeneity and potential bias. Furthermore, extremely high performance estimates reported in isolated studies may reflect small sample sizes, limited external validation, or potential overfitting, and therefore warrant cautious interpretation. The quality assessment supported these observations, identifying retrospective patient selection, lack of external validation, heterogeneous reference standards, and incomplete reporting of model development and validation procedures as the most common methodological limitations across the included studies. Importantly, these methodological limitations were consistent across the majority of studies and reflect the current developmental stage of AI research in bone metastases rather than isolated deficiencies of individual investigations. Future studies should prioritize prospective multicenter validation, standardized reporting of model development and calibration, and independent external validation to improve methodological robustness and facilitate clinical translation.

Looking forward, meaningful clinical translation will depend not only on maintaining high discriminative performance but also on demonstrating robustness across diverse populations and imaging platforms, ensuring interpretability, and establishing measurable improvements in patient outcomes. Beyond diagnostic performance, successful implementation will also require standardized reporting, interoperability with clinical imaging workflows, regulatory approval, and demonstration of reproducible clinical benefit across diverse healthcare settings. Only through such rigorous validation can AI evolve from promising research methodology to reliable clinical decision-support tool. Current evidence suggests that radiomics-based imaging models and AI-assisted nuclear medicine applications are the most mature areas for potential clinical translation. Conversely, multimodal AI models integrating imaging with pathological or genomic information, large language models, and generative AI applications remain investigational and require substantially more prospective validation before routine implementation.

## Conclusions

Artificial intelligence–based models demonstrate high diagnostic performance for bone metastases across diverse imaging modalities and analytical approaches. Radiomics and imaging-based deep learning methods show particularly consistent results when evaluated within homogeneous methodological categories. Nevertheless, substantial methodological heterogeneity and limited prospective validation currently preclude immediate routine clinical implementation. Future research should prioritize robust external validation and outcome-driven evaluation to define the role of AI in routine bone oncology practice (34). At present, AI should be regarded as a clinical decision-support tool rather than an autonomous diagnostic system. Its routine implementation will require prospective multicenter validation, standardized reporting, regulatory evaluation, and demonstration of measurable clinical benefit across diverse healthcare settings.

## Data Availability

The original contributions presented in the study are included in the article/**Supplementary Material**. Further inquiries can be directed to the corresponding author.
