# Supplementary material for: Artificial intelligence for bone metastases: a systematic review and meta-analysis of diagnostic and prognostic performance
Source: Front Oncol. 2026 Aug 24;16:1915919. doi: 10.3389/fonc.2026.1915919 (PMC13547069; doi:10.3389/fonc.2026.1915919)
Supplement: Supplementary file 1 [file Table1.docx]

**Supplementary Table S1. Search strategies used for the literature search**

**PubMed/MEDLINE**

(("Bone Neoplasms"[Mesh] OR "bone metastasis" OR "bone metastases" OR "skeletal metastasis" OR "skeletal metastases")

AND

("Artificial Intelligence"[Mesh] OR "artificial intelligence" OR "machine learning" OR "deep learning" OR radiomics))

AND

(diagnos* OR detect* OR prognos* OR predict*)

**Scopus**

TITLE-ABS-KEY

(

("bone metastasis" OR "bone metastases" OR "skeletal metastasis" OR "skeletal metastases")

AND

("artificial intelligence" OR "machine learning" OR "deep learning" OR radiomics)

AND

(diagnos* OR detect* OR prognos* OR predict*)

)

**Web of Science Core Collection**

TS=

(

("bone metastasis" OR "bone metastases" OR "skeletal metastasis" OR "skeletal metastases")

AND

("artificial intelligence" OR "machine learning" OR "deep learning" OR radiomics)

AND

(diagnos* OR detect* OR prognos* OR predict*)

)
